# First record of Eggplant Mealybug, *Coccidohystrix insolita* (Hemiptera: Pseudococcidae), on Guam: Potentially a major pest

**DOI:** 10.3897/BDJ.2.e1042

**Published:** 2014-01-23

**Authors:** Aubrey Moore, Gillian W. Watson, Jesse Bamba

**Affiliations:** † Western Pacific Tropical Research Center, University of Guam, Mangilao, Guam, United States of America; ‡ California Department of Food and Agriculture, Sacramento, California, United States of America; § Cooperative Extension Service, University of Guam, Mangilao, Guam, United States of America

**Keywords:** *Coccidohystrix insolita*, eggplant mealybug, invasive species

## Abstract

The eggplant mealybug, *Coccidohystrix insolita* (Green) (Hemiptera: Pseudococcidae), is recorded from the island of Guam in the Mariana Islands for the first time. Factors indicating that this introduced mealybug has the potential to become a pest of economic importance for agriculture and horticulture on Guam are discussed.

## Introduction

The eggplant mealybug, *Coccidohystrix insolita* (Green) is broadly distributed in the tropics and subtropics and well known as a agricultural and horticultural pest (Lit et al. 1998, Williams 2004, Williams and Watson 1988). This article documents the first detection of *Coccidohystrix insolita* on Guam.

**Figure 1.**
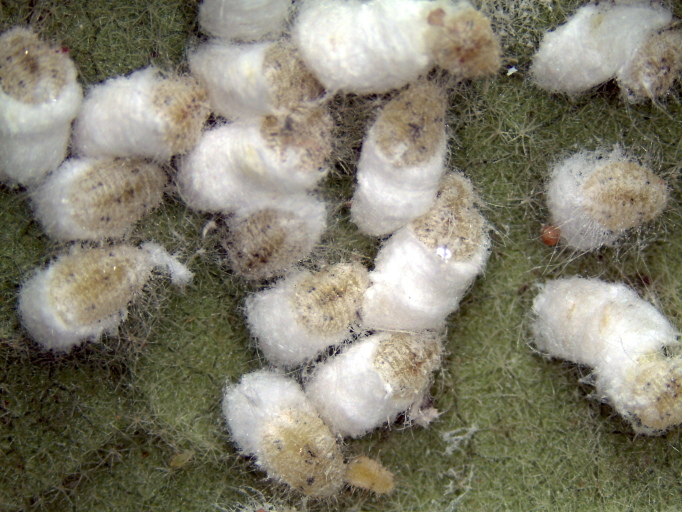
Adult *Coccidohystrix insolita* females with ovisacs.

**Figure 2.**
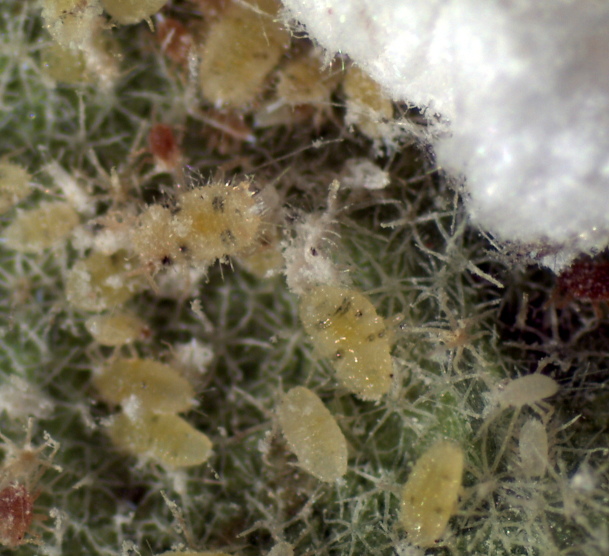
*Coccidohystrix insolita* nymphs.

**Figure 3.**
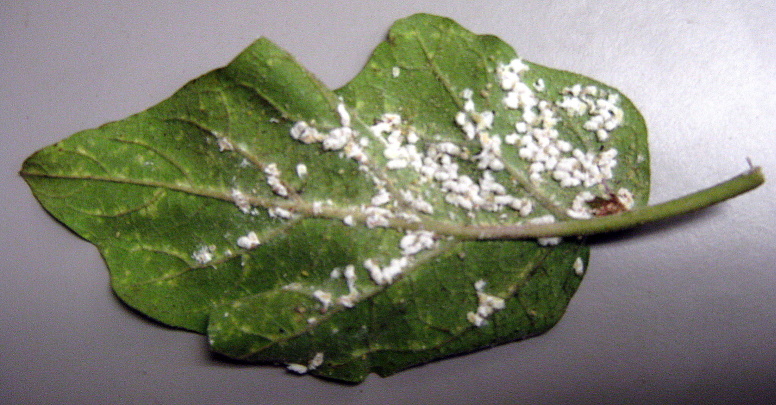
Eggplant (*Solanum melongena* L.) leaf underside infested with *Coccidohystrix insolita*.

Mealybugs producing long ovisacs were found infesting the lower leaf surfaces of eggplant, *Solanum melongena* L., in a farmer's field on Guam on December 4, 2013 (Figs 1, 2, 3). Samples of infested leaves were preserved in ethanol for subsequent identification at the California Department of Food and Agriculture Diagnostic Center in Sacramento, California. Diagnosis was based on morphology of adult females mounted on microscope slides.

## Materials and methods

Infested leaves were collected into bags and taken to the laboratory. Color photographs were taken to record the appearance of the insects in life using a Leica EZ4HD dissection microscope. Infested leaf fragments were preserved in 70% ethanol and sent to the Plant Pest Diagnostic laboratory of California Department of Food and Agriculture (CDFA-PPDC) for diagnosis. A total of seven adult female specimens were selected and prepared on three microscope slides using the method given by Sirisena et al. 2013. The specimens were studied using a Nikon Eclipse 80i compound microscope with phase contrast illumination and ×30 – ×600 magnification, and were identified using the keys in Williams and Watson 1988 and Williams 2004. Slide-mounted voucher specimens will be deposited in the California State Arthropod Collection at CDFA-PPDC in Sacramento, California.

## Taxon treatments

### Coccidohystrix insolita

(Green, 1908)

#### Materials

- **Type status:** Other material. **Occurrence:** catalogNumber: AM20131204.002; occurrenceRemarks: on eggplant leaves; recordedBy: Jesse Bamba; sex: 7 slide-mounted adult females were examined; **Location:** islandGroup: Mariana Islands; island: Guam; municipality: Dededo; locality: near Swamp Road; decimalLatitude: 13.539981; decimalLongitude: 144.83435; **Identification:** identifiedBy: Gillian W. Watson; dateIdentified: 2013-12-13; **Event:** samplingProtocol: eggplant leaf samples; eventDate: 2013-12-04; **Record Level:** collectionID: ESUG; institutionCode: UGUAM; basisOfRecord: LivingSpecimen; source: http://guaminsects.myspecies.info/node/2623

#### Diagnosis

9-segmented; posterior ostioles present, anterior ostioles absent; cerarii on margins numbering 17 pairs, numerous dorsal cerarii present also, each cerarius consisting of 1–15 large conical setae situated on a sclerotized prominence, without any associated trilocular pores; legs well developed, each claw with a denticle present on plantar surface; circulus absent; anal lobes well developed, each with a sclerotized ventral bar; quinquelocular pores numerous on venter; multilocular disc pores numerous on venter of abdominal segments III-IX, a few also present on the venter of segments I and II and on the dorsum of segment VII; ventral oral collar ducts present on submargins of abdominal segments V-VIII; oral rim ducts absent entirely.

Diagnosis was based Williams 2004 which includes a good taxonomic illustration of *Coccidohystrix insolita*.

#### Distribution

*Coccidohystrix insolita* has been recorded in the literature from the following regions and countries:

**Afrotropical:** Kenya, Madagascar, Rodriques Island (Mauritius), South Africa, Tanzania, Zanzibar; **Australasian**: Western Samoa; **Oriental**: Bangladesh, Burma (=Myanmar), India, Laos, Pakistan, Philippines, Sri Lanka, Thailand, Vietnam; **Palaearctic**: China, Saudi Arabia (Ben-Dov 2013).

In addition, a Japanese quarantine inspector found *Coccidohystrix insolita* on *Alternanthera* (Amaranthaceae) imported from Singapore (Tokihiro 2006).

Prior to our discovery on Guam, *Coccidohystrix insolita* was known only from two Pacific island nations: the Philippines and Western Samoa. *Coccidohystrix insolita* was first detected in the Philippines during 1994 (Lit et al. 1998) and in Western Samoa in 1966 (Williams and Watson 1988).

#### Biology

*Coccidohystrix insolita* lives on the leaves (Fig. 3).

#### Notes

The appearance of *Coccidohystrix insolita* in life is unusual for a mealybug because the adult female has very little dorsal wax and secretes a white, waxy ovisac up to 6 times as long as the body of the female (Fig. 1), which is more typical of some Coccidae. The immature stages do not secrete a thick layer of mealy wax, the body being shiny yellow-green with submedian grey spots on 2 abdominal and 1 thoracic segments (Fig. 2). This contrasts with the in life appearance of the solenopsis mealybug, *Phenacoccus solenopsis* Tinsley, in which all developmental stages develop a thick layer of white mealy wax except for two longitudinal lines of bare cuticle that expose dark submedian spots on 3 or 4 segments on the abdomen and 1 or 2 on the thorax.

#### Host Plants

*Coccidohystrix insolita* is polyphagous and is recorded from the following families of host plants (Ben-Dov 2013): Acanthaceae, Amaranthaceae, Apocynaceae, Araceae, Arecaceae, Aristolochiaceae, Asteraceae, Chenopodiaceae, Cucurbitaceae, Euphorbiaceae, Fabaceae, Malvaceae, Menispermaceae, Moraceae, Poaceae, Rhamnaceae, Rubiaceae, Solanaceae, Sterculiaceae, Tiliaceae, Zygophyllaceae.

Many plants belonging to these families are important to agriculture and forestry on Guam.

#### Parasitoids

Twenty-three species of hymenopterous parasitoids are associated with *Coccidohystrix insolita* (Noyes 2013):

**Aphelinidae:** *Coccophagus pseudococci*; **Encyrtidae:** *Adektitopus longipennis*, *Anagyrus gracilis*, *Apoleptomastix bicoloricornis*, *Blepyrus insularis*, *Gyranusoidea signata*, *Homalotylus albiclavatus*, *Homalotylus hemipterinus*, *Homalotylus indicus*, *Homalotylus turkmenicus*, *Leptomastix nigrocincta*, *Leptomastix nigrocoxalis*, *Neocharitopus orientalis*, *Paranathrix tachikawai*, *Prochiloneurus albifuniculus*, *Prochiloneurus pulchellus*; **Eulophidae:** *Aprostocetus ajmerensis*, *Aprostocetus annulicornis*, *Aprostocetus jaipurensis*; **Pteromalidae:** *Catolaccus crassiceps*; **Signiphoridae:** *Chartocerus hyalipennis*, *Chartocerus kerrichi*, *Chartocerus kurdjumovi*.

None of these species are known to exist on Guam and there were no signs of parasitism in the specimens examined.

#### Other Natural Enemies

The following natural enemies have been recorded attacking *Coccidohystrix insolita*: **Fungi:** *Metarhizium anisopliae*; **Insecta: Coleoptera: Coccinellidae:** *Anegleis cardoni* (Weise); *Hyperaspis maindronia*; *Nephus regularis*; **Lepidoptera:** Lycaenidae: *Spalgis epeus* (Ben-Dov 2013). None of the insect predators are known to exist on Guam.

#### Attendant Ants

Three species of attendant ants are associated with *Coccidohystrix insolita*: Dolichoderus bituberculatus, *Solenopsis geminata*, *Anoplolepis gracilipes* (Ben-Dov 2013). The latter two species are abundant on Guam but so far, we have not yet seen any ant associations with *Coccidohystrix insolita*.

## Discussion

Guam, like all small tropical islands, is susceptible to damage from invasive species because of a warm climate with no winter, coupled with a lack of natural enemies for many new arrivals. It is difficult to predict the eventual pest status of any newly detected invasive insect species, but *Coccidohystrix insolita* has the hallmarks of being a major pest on Guam for several reasons:

1. **Plant hosts.** Many of the known host plants of *Coccidohystrix insolita* are commonly grown as crops on Guam. In addition several others, such as coconut palm, *Cocos nucifera*, and *Hibiscus* spp. are major components of natural and ornamental vegetation.
2. **Escape from natural enemies.** None of the parasitoids or predators known to attack *Coccidohystrix insolita* are known to exist on Guam. Despite the fact that several parasitoids and predators pre-existed in the Philippines prior to arrival of *Coccidohystrix insolita*, this species became a major agricultural pest (Lit et al. 1998). It is likely that implementation of biological control will be required to prevent major economic and environmental damage by this pest on Guam.
3. **Attendant ants.** Two of the three ants known to form a commensal relationship with *Coccidohystrix insolita*, namely *Anoplolepis gracilipes* and *Solenopsis geminata*, are common on Guam. In addition, several other ant species which readily establish associations with mealybugs are present. Attendant ants protect mealybugs from parasitism and predation, making it difficult to establish biological control.
4. **Origin.** Guam is an unincorporated territory of the United States of America (U.S.). Experience has shown that invasive species which originate from outside of the U.S., such as this one, are harder to deal with than those accidentally imported from the U.S. mainland or Hawaii. For invasive insect species already present in the U.S., control resources are usually readily available. Often research has been done, control methods have been developed, biological control agents have been identified, an exploratory entomologist has been sent out to collect candidate species, and these have been imported, cultured and tested, and are available for use on Guam. However, resources are scant when it comes to responding to invasive species of non-U.S. origin.
5. **Rapid Response Capacity.** There is currently a critical lack of capacity to deal with entomological problems on Guam and in the rest of Micronesia. The number of Ph.D. level entomologists practicing on Guam and in the rest of Micronesia has decreased from nine during the mid-1990s to only three at present.
6. **Biological Control Agent Import Permits.** Guam is required to comply with U.S. Department of Agriculture regulations for importing biological control agents. These requirements are far more stringent for organisms originating outside of the U.S. than for those imported from within the U.S. Delays in the permitting process and a lack of capacity to comply with permit conditions sometimes impede rapid progress towards establishment of biological control in time to prevent major economic and environmental damage. Often, there is a pest population explosion prior to implementation of biological control. During this initial outbreak, risk of accidental export to trading partners is high.

## Supplementary Material

XML Treatment for Coccidohystrix insolita
